# Supplementary material for: End Sequence Analysis Toolkit (ESAT) expands the extractable information from single-cell RNA-seq data
Source: Genome Res. 2016 Oct;26(10):1397–410. doi: 10.1101/gr.207902.116 (PMC5052061; doi:10.1101/gr.207902.116)
Supplement: Supplemental Material [file supp_gr.207902.116_Supp_Materials.tar.gz › ESAT-esat_v0.1/doc/html/classbroad_1_1core_1_1motif_1_1_position_weight_matrix.html]

ESAT: broad.core.motif.PositionWeightMatrix Class Reference


ESAT


- Main Page
- Related Pages
- Packages
- Classes
- Files

- Class List
- Class Index
- Class Hierarchy
- Class Members

All Classes Namespaces Files Functions Variables Enumerator Pages

- broad
- core
- motif
- PositionWeightMatrix

Public Member Functions |
Static Public Member Functions |
Protected Member Functions |
List of all members

broad.core.motif.PositionWeightMatrix Class Reference

Inheritance diagram for broad.core.motif.PositionWeightMatrix:

[legend]

Collaboration diagram for broad.core.motif.PositionWeightMatrix:

[legend]

|  |  |
| --- | --- |
| Public Member Functions | |
|  | PositionWeightMatrix (String name) |
|  | |
| void | setMatrixFromRawData (List< String[]> unparsedRows) throws ParseException |
|  | |
| void | setMatrix (List< double[]> sites) throws ParseException |
|  | |
| void | addColumn (double[] site) |
|  | |
| void | setUnparsedRow (int colNum, String[] unparsedRow) throws ParseException |
|  | |
| void | write (BufferedWriter bw, NumberFormat formatter) throws IOException |
|  | |
| void | writeUNIPROBE (BufferedWriter bw, NumberFormat formatter) throws IOException |
|  | |
| String | getName () |
|  | |
| void | setName (String name) |
|  | |
| int | getLeftHighInfoStart () |
|  | |
| int | getRightHighInfoStart () |
|  | |
| void | setAlignPos (int minAlignNum) |
|  | |
| PositionWeightMatrix | trimByInformationContent (double ic) |
|  | |
| List< Double > | computeScoreDistribution (float backgroundA, float backgroundC, float backgroundG, float backgroundT, int sampleSize) |
|  | |
| String | printKmer (int[] kmer) |
|  | |
| double | getLogLikelihood (char[] s) |
|  | |
| double | getLogLikelihood (int[] s) |
|  | |
| double | getLogLikelihood (short[] encodedSequence, int i) |
|  | |
| int | getNumCol () |
|  | |
| boolean | isEmpty () |
|  | |
| PositionWeightMatrix | permuteColumns (boolean preserveGCDinucleotides) |
|  | |
| PositionWeightMatrix | copy () |
|  | |
| double | ic () |
|  | |
| PositionWeightMatrix | reverseComplement () |
|  | |
| int | getNumEndPosToErase (double infoThreshold) |
|  | |
| int | getNumStartPosToErase (double infoThreshold) |
|  | |
| void | printRefinedWeightMatrix (double infoThreshold) |
|  | |
| void | flatWeightMatrix (double infoTh) |
|  | |
| String | getConsensus () |
|  | |
| boolean | getAlignDir () |
|  | |
| List< BED > | match (SequenceRegion region, double[] background, float minScore) |
|  | |
| PositionWeightMatrix | createIsoPWM (double[] column, String name) |
|  | |
| void | addPseudoCounts () |
|  | |
| PositionWeightMatrix | centroidOf (Collection< PositionWeightMatrix > pwmSet) throws IllegalArgumentException |
|  | |
| double | distanceFrom (PositionWeightMatrix other) |
|  | |
| double | kullbackLeiber (PositionWeightMatrix other) |
|  | |

|  |  |
| --- | --- |
| Static Public Member Functions | |
| static int[] | getNextKmer (int[] kmer) |
|  | |

|  |  |
| --- | --- |
| Protected Member Functions | |
| void | addToCentroid (Matrix m, PositionWeightMatrix pwm) |
|  | |

## Constructor & Destructor Documentation

|  |  |  |  |  |  |
| --- | --- | --- | --- | --- | --- |
| broad.core.motif.PositionWeightMatrix.PositionWeightMatrix | ( | String | *name* | ) |  |

Here is the caller graph for this function:

## Member Function Documentation

|  |  |  |  |  |  |
| --- | --- | --- | --- | --- | --- |
| void broad.core.motif.PositionWeightMatrix.addColumn | ( | double[] | *site* | ) |  |

|  |  |  |  |  |
| --- | --- | --- | --- | --- |
| void broad.core.motif.PositionWeightMatrix.addPseudoCounts | ( |  | ) |  |

|  |  |  |  |
| --- | --- | --- | --- |
| void broad.core.motif.PositionWeightMatrix.addToCentroid | ( | Matrix | *m*, |
|  |  | PositionWeightMatrix | *pwm* |
|  | ) |  |  |

protected

Here is the call graph for this function:

Here is the caller graph for this function:

|  |  |  |  |  |  |
| --- | --- | --- | --- | --- | --- |
| PositionWeightMatrix broad.core.motif.PositionWeightMatrix.centroidOf | ( | Collection< PositionWeightMatrix > | *pwmSet* | ) | throws IllegalArgumentException |

Computes the euclidean centroid, it may not be the formal centroid for different metrics but intuitively the average counts should provide a good cluster representative which is what this method intends to return

Parameters
:   |  |  |
    | --- | --- |
    | pwmSet | - Collection of pwms from which to compute the centroid0 |

Returns
:   The euclidean centroid

Exceptions
:   |  |  |
    | --- | --- |
    | IllegalArgumentException | - When not all PWMs have the same dimension. |

Here is the call graph for this function:

|  |  |  |  |
| --- | --- | --- | --- |
| List<Double> broad.core.motif.PositionWeightMatrix.computeScoreDistribution | ( | float | *backgroundA*, |
|  |  | float | *backgroundC*, |
|  |  | float | *backgroundG*, |
|  |  | float | *backgroundT*, |
|  |  | int | *sampleSize* |
|  | ) |  |  |

Compute Distribution of scores for motif.

Parameters
:   |  |  |
    | --- | --- |
    | s |  |
    | isNumSeq |  |

Returns

Here is the call graph for this function:

|  |  |  |  |  |
| --- | --- | --- | --- | --- |
| PositionWeightMatrix broad.core.motif.PositionWeightMatrix.copy | ( |  | ) |  |

Here is the call graph for this function:

Here is the caller graph for this function:

|  |  |  |  |
| --- | --- | --- | --- |
| PositionWeightMatrix broad.core.motif.PositionWeightMatrix.createIsoPWM | ( | double[] | *column*, |
|  |  | String | *name* |
|  | ) |  |  |

Creates a PWM of similar dimension to this PWM but with all columns set to the given vector, usually a neutral mutation vector.

Parameters
:   |  |  |
    | --- | --- |
    | column |  |

Returns

Here is the call graph for this function:

Here is the caller graph for this function:

|  |  |  |  |  |  |
| --- | --- | --- | --- | --- | --- |
| double broad.core.motif.PositionWeightMatrix.distanceFrom | ( | PositionWeightMatrix | *other* | ) |  |

Distance between two PWMs, uses the KL similarity

Here is the call graph for this function:

|  |  |  |  |  |  |
| --- | --- | --- | --- | --- | --- |
| void broad.core.motif.PositionWeightMatrix.flatWeightMatrix | ( | double | *infoTh* | ) |  |

|  |  |  |  |  |
| --- | --- | --- | --- | --- |
| boolean broad.core.motif.PositionWeightMatrix.getAlignDir | ( |  | ) |  |

|  |  |  |  |  |
| --- | --- | --- | --- | --- |
| String broad.core.motif.PositionWeightMatrix.getConsensus | ( |  | ) |  |

|  |  |  |  |  |
| --- | --- | --- | --- | --- |
| int broad.core.motif.PositionWeightMatrix.getLeftHighInfoStart | ( |  | ) |  |

|  |  |  |  |  |  |
| --- | --- | --- | --- | --- | --- |
| double broad.core.motif.PositionWeightMatrix.getLogLikelihood | ( | char[] | *s* | ) |  |

Here is the caller graph for this function:

|  |  |  |  |  |  |
| --- | --- | --- | --- | --- | --- |
| double broad.core.motif.PositionWeightMatrix.getLogLikelihood | ( | int[] | *s* | ) |  |

|  |  |  |  |
| --- | --- | --- | --- |
| double broad.core.motif.PositionWeightMatrix.getLogLikelihood | ( | short[] | *encodedSequence*, |
|  |  | int | *i* |
|  | ) |  |  |

|  |  |  |  |  |
| --- | --- | --- | --- | --- |
| String broad.core.motif.PositionWeightMatrix.getName | ( |  | ) |  |

Here is the caller graph for this function:

|  |  |  |  |  |  |
| --- | --- | --- | --- | --- | --- |
| static int [] broad.core.motif.PositionWeightMatrix.getNextKmer | ( | int[] | *kmer* | ) |  |

static

Gets the next logical kmer. If k = 4 the first 5 4mers would be: (0,0,0,0), (0,0,0,1), (0,0,0,2), (0,0,0,3), (0,0,1,0).

Parameters
:   |  |  |
    | --- | --- |
    | kmer | - any valid kmer |

Returns
:   Next logical kmer.

|  |  |  |  |  |
| --- | --- | --- | --- | --- |
| int broad.core.motif.PositionWeightMatrix.getNumCol | ( |  | ) |  |

Here is the caller graph for this function:

|  |  |  |  |  |  |
| --- | --- | --- | --- | --- | --- |
| int broad.core.motif.PositionWeightMatrix.getNumEndPosToErase | ( | double | *infoThreshold* | ) |  |

Here is the caller graph for this function:

|  |  |  |  |  |  |
| --- | --- | --- | --- | --- | --- |
| int broad.core.motif.PositionWeightMatrix.getNumStartPosToErase | ( | double | *infoThreshold* | ) |  |

Here is the caller graph for this function:

|  |  |  |  |  |
| --- | --- | --- | --- | --- |
| int broad.core.motif.PositionWeightMatrix.getRightHighInfoStart | ( |  | ) |  |

|  |  |  |  |  |
| --- | --- | --- | --- | --- |
| double broad.core.motif.PositionWeightMatrix.ic | ( |  | ) |  |

Computes the information content of this position weith matris

Returns

|  |  |  |  |  |
| --- | --- | --- | --- | --- |
| boolean broad.core.motif.PositionWeightMatrix.isEmpty | ( |  | ) |  |

|  |  |  |  |  |  |
| --- | --- | --- | --- | --- | --- |
| double broad.core.motif.PositionWeightMatrix.kullbackLeiber | ( | PositionWeightMatrix | *other* | ) |  |

Here is the caller graph for this function:

|  |  |  |  |
| --- | --- | --- | --- |
| List<BED> broad.core.motif.PositionWeightMatrix.match | ( | SequenceRegion | *region*, |
|  |  | double[] | *background*, |
|  |  | float | *minScore* |
|  | ) |  |  |

Here is the call graph for this function:

|  |  |  |  |  |  |
| --- | --- | --- | --- | --- | --- |
| PositionWeightMatrix broad.core.motif.PositionWeightMatrix.permuteColumns | ( | boolean | *preserveGCDinucleotides* | ) |  |

Here is the call graph for this function:

|  |  |  |  |  |  |
| --- | --- | --- | --- | --- | --- |
| String broad.core.motif.PositionWeightMatrix.printKmer | ( | int[] | *kmer* | ) |  |

|  |  |  |  |  |  |
| --- | --- | --- | --- | --- | --- |
| void broad.core.motif.PositionWeightMatrix.printRefinedWeightMatrix | ( | double | *infoThreshold* | ) |  |

Here is the call graph for this function:

|  |  |  |  |  |
| --- | --- | --- | --- | --- |
| PositionWeightMatrix broad.core.motif.PositionWeightMatrix.reverseComplement | ( |  | ) |  |

Here is the call graph for this function:

|  |  |  |  |  |  |
| --- | --- | --- | --- | --- | --- |
| void broad.core.motif.PositionWeightMatrix.setAlignPos | ( | int | *minAlignNum* | ) |  |

|  |  |  |  |  |  |
| --- | --- | --- | --- | --- | --- |
| void broad.core.motif.PositionWeightMatrix.setMatrix | ( | List< double[]> | *sites* | ) | throws ParseException |

|  |  |  |  |  |  |
| --- | --- | --- | --- | --- | --- |
| void broad.core.motif.PositionWeightMatrix.setMatrixFromRawData | ( | List< String[]> | *unparsedRows* | ) | throws ParseException |

Here is the call graph for this function:

|  |  |  |  |  |  |
| --- | --- | --- | --- | --- | --- |
| void broad.core.motif.PositionWeightMatrix.setName | ( | String | *name* | ) |  |

|  |  |  |  |
| --- | --- | --- | --- |
| void broad.core.motif.PositionWeightMatrix.setUnparsedRow | ( | int | *colNum*, |
|  |  | String[] | *unparsedRow* |
|  | ) |  | throws ParseException |

Here is the caller graph for this function:

|  |  |  |  |  |  |
| --- | --- | --- | --- | --- | --- |
| PositionWeightMatrix broad.core.motif.PositionWeightMatrix.trimByInformationContent | ( | double | *ic* | ) |  |

Trims this PWM by removing start end ending columns with information content lesser than given.

Parameters
:   |  |  |
    | --- | --- |
    | ic | - Columns below this ic at PWM edges will be trimmed. |

Returns
:   The resulting PWM.

Here is the call graph for this function:

|  |  |  |  |
| --- | --- | --- | --- |
| void broad.core.motif.PositionWeightMatrix.write | ( | BufferedWriter | *bw*, |
|  |  | NumberFormat | *formatter* |
|  | ) |  | throws IOException |

|  |  |  |  |
| --- | --- | --- | --- |
| void broad.core.motif.PositionWeightMatrix.writeUNIPROBE | ( | BufferedWriter | *bw*, |
|  |  | NumberFormat | *formatter* |
|  | ) |  | throws IOException |

---

The documentation for this class was generated from the following file:

- java/broad/core/motif/PositionWeightMatrix.java


---

Generated on Fri Aug 15 2014 15:10:54 for ESAT by  

 1.8.7
